# Author Correction: Global methane emissions from rivers and streams

**DOI:** 10.1038/s41586-025-09130-8

**Published:** 2025-05-22

**Authors:** Gerard Rocher-Ros, Emily H. Stanley, Luke C. Loken, Nora J. Casson, Peter A. Raymond, Shaoda Liu, Giuseppe Amatulli, Ryan A. Sponseller

**Affiliations:** 1 Department of Ecology and Environmental Science, Umeå University, Umeå, Sweden; 2 Department of Forest Ecology and Management, Swedish University of Agricultural Sciences, Umeå, Sweden; 3 Integrative Freshwater Ecology Group, Centre for Advanced Studies of Blanes (CEAB-CSIC), Blanes, Spain; 4 Center for Limnology, University of Wisconsin–Madison, Madison, WI USA; 5 Upper Midwest Water Science Center, United States Geological Survey, Madison, WI USA; 6 Department of Geography, University of Winnipeg, Winnipeg, Manitoba Canada; 7 School of the Environment, Yale University, New Haven, CT USA; 8 State Key Laboratory of Water Environment Simulation, School of Environment, Beijing Normal University, Beijing, China

**Keywords:** Carbon cycle, Limnology, Ecosystem ecology, Hydrology

Correction to: *Nature* 10.1038/s41586-023-06344-6 Published online 16 August 2023

In the version of the article initially published, the dashed black line in Fig. 4a, representing the slope from the average activation energy of other aquatic systems, was incorrect and has now been amended in the HTML and PDF versions of the article, as seen in Fig. 1.

**Fig. 1:**
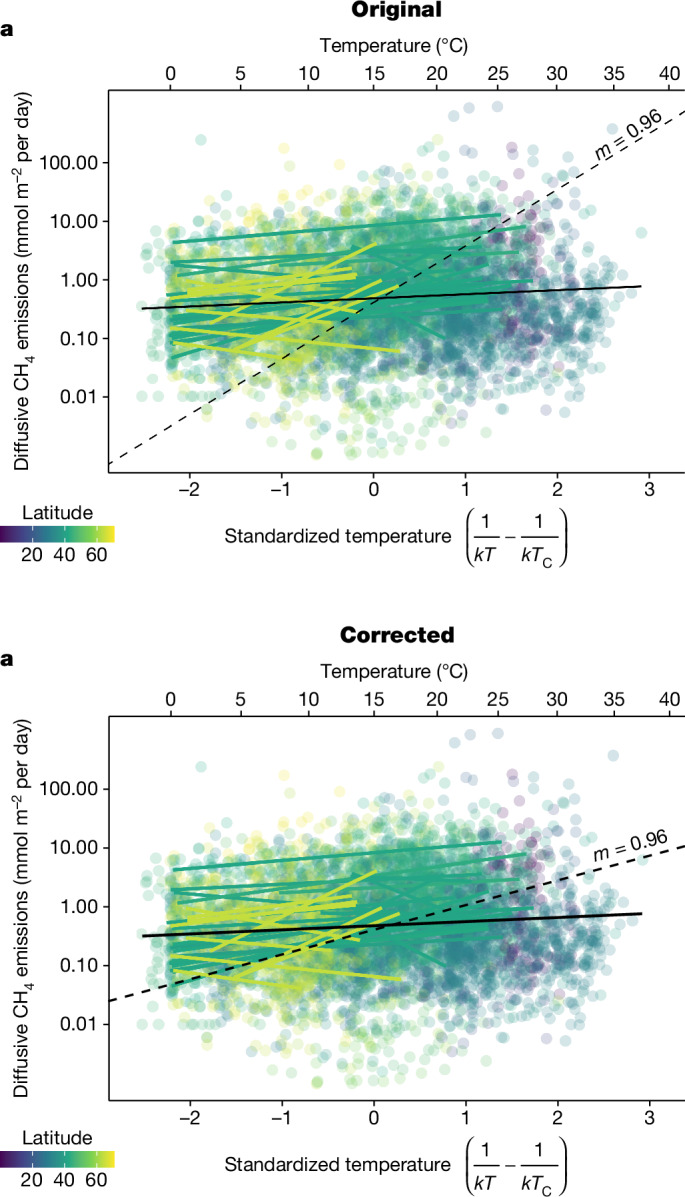
Original and corrected Fig. 4a .

