# Supplementary material for: Global methane emissions from rivers and streams
Source: Nature. 2023 Aug 16;621(7979):530–5. doi: 10.1038/s41586-023-06344-6 (PMC10511311; doi:10.1038/s41586-023-06344-6)
Supplement: Supplementary file 1 — Supplementary Information [file 41586_2023_6344_MOESM1_ESM.pdf]

nature portfolio

https://doi.org/10.1038/s41586-023-06344-6

Supplementary information

Global methane emissions from rivers and streams

In the format provided by the authors and unedited

Supplementary Materials for

Global methane emissions from rivers and streams

Gerard Rocher-Ros[1,2,3]*, Emily H. Stanley[4], Luke C. Loken[5], Nora J. Casson[6], Peter A. Raymond[7], Shaoda Liu[7,8], Giuseppe Amatulli[7], Ryan A. Sponseller[1]

[1] Department of Ecology and Environmental Science, Umeå University, 90187, Sweden

[2] Department of Forest Ecology and Management, Swedish University of Agricultural Sciences, 90183 Umeå, Sweden

[3] Integrative Freshwater Ecology Group, Centre for Advanced Studies of Blanes (CEAB-CSIC), 17300 Blanes, Spain

[4] Center for Limnology, University of Wisconsin-Madison, Madison, WI, USA

[5] U.S. Geological Survey, Upper Midwest Water Science Center, Madison, WI, USA

[6] Department of Geography, University of Winnipeg, Winnipeg MB, Canada

[7] School of the Environment, Yale University, New Haven, CT 06511, USA

[8] State Key Laboratory of Water Environment Simulation, School of Environment, Beijing Normal University, Beijing, 100875 China

*Correspondence to: gerard.rocher.ros@slu.se

Table of contents:

Supplementary methods: Assessment of other machine learning models……….……………2

Figure S6. Partial dependence plots…………………….……………………………………. 3

Table S1. Summary characteristics of GRADES river reaches...……………………………..….4

Table S2. Spatial predictors used to predict methane concentrations…………………………5

Table S3. Variance partitions about drivers of riverine methane emissions…..………………6

Table S4. Monthly model performance of the three machine-learning algorithms tested…….6

Supplementary methods: Assessment of other machine learning models

In addition to the random forest models presented in the main text, we investigated whether other machine learning algorithms more oriented towards predictability could improve the results with this dataset. Those methods could in principle provide a more robust prediction and can also be interpreted similarly to random forest models. Specifically, we used a gradient boosting algorithm "XGBoost" and a single-layer feed-forward neural network to consider this possibility. All models were implemented using yearly averages of all values in each river reach. Gradient boosting was implemented using "XGBoost" after a grid search to find the optimal combination of the parameters. We also tested a single layer, feedforward neural network model, with 10 hidden units, implemented using "*nnet*". More details on the hyperparameters used and R code are available in the script "2_model_selection.R" in github: (https://github.com/rocher-ros/RiverMethaneFlux).

Overall, we found that the performance of the three algorithms was similar, with marginally better predictive ability from the random forest model compared the others. Interestingly, when the same models were implemented on the monthly datasets, the random forest model and the gradient boosting algorithms performs substantially better compared to the yearly dataset, while the neural network model underperformed markedly (See Table S4 for a monthly summary of key statistics).

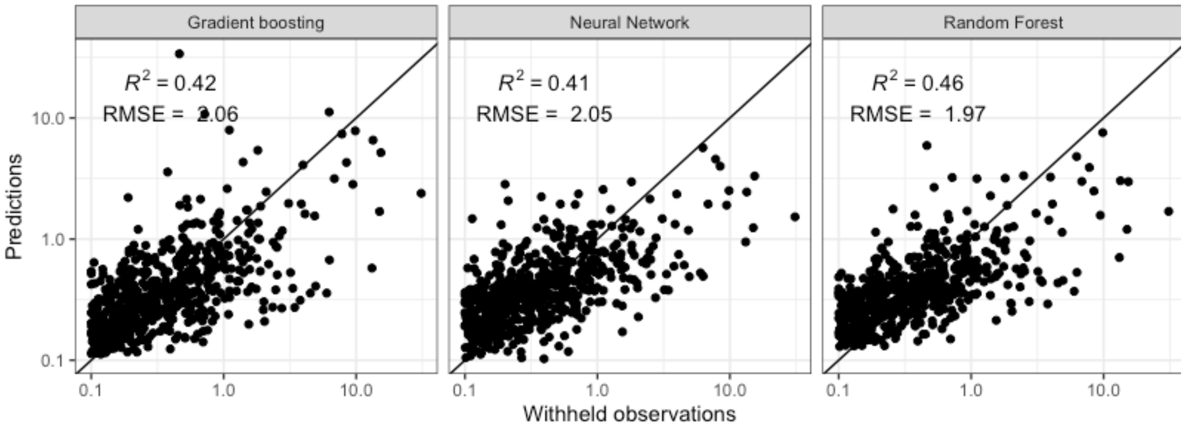

Figure S1. Performance of the three machine-learning algorithms tested predicting methane concentrations, including the random forest model used in the main text. The x-axis are observations in the dataset withheld to the model, and in the y-axis the predicted values for each method, with the R^2 and Root Mean Square Error in the plot.

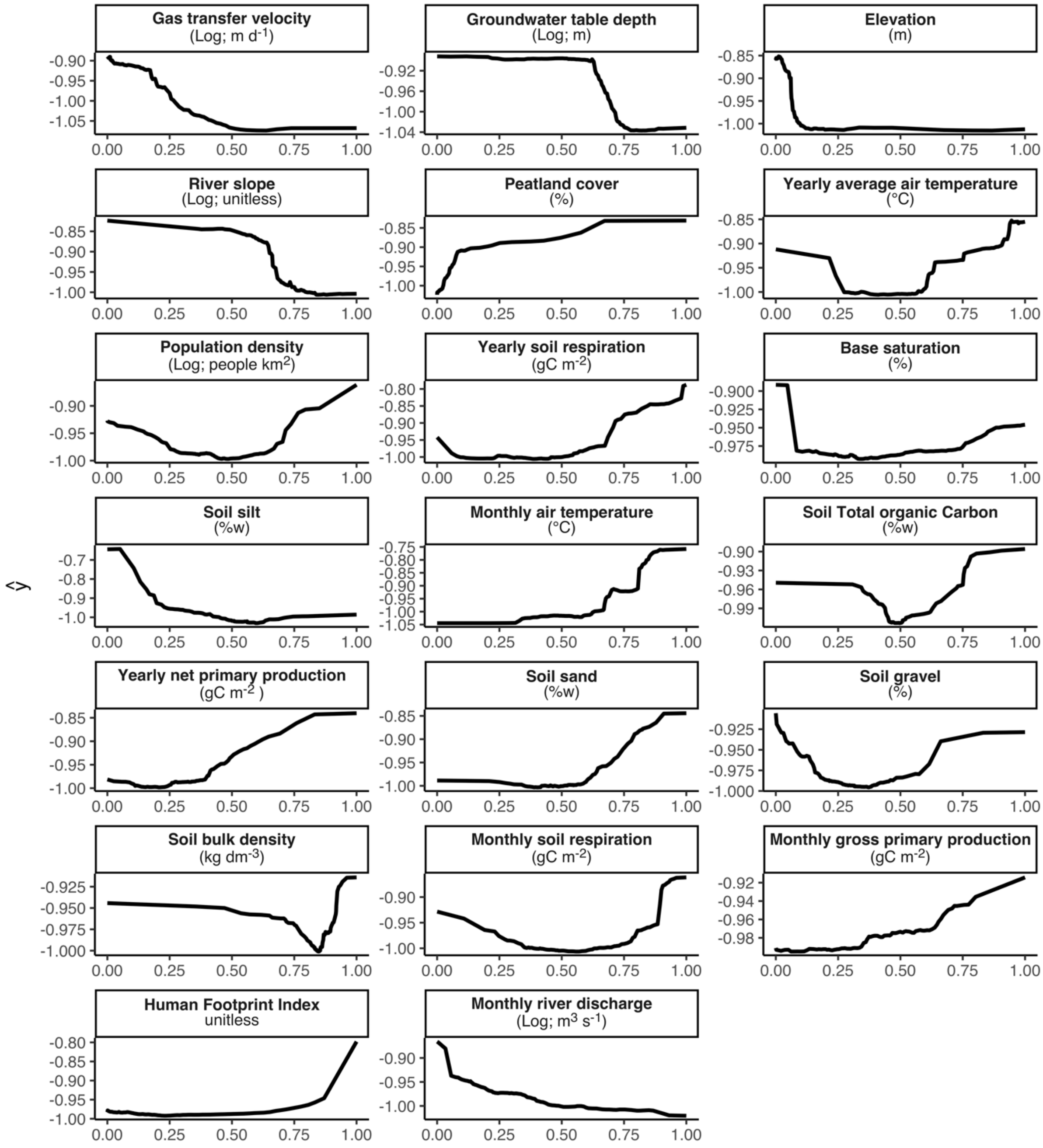

Figure S2: Partial dependence plots. The marginal effect of a given variable on the predicted methane concentrations (ŷ) for the 20 most important variables considered. Each of those plots are shown as bar insets in Figure 2, but values three were all rescaled to the same range for a better visualization.

Table S1: Summary characteristics of Global Reach-level A priori Discharge Estimates (GRADES[1]) river reaches. Median values are reported for width, length, and slope.

Stream order	n	Width (m)	Length (m)	Slope (unitless)
1	1,478,622	4.49	6,517	0.00881
2	696,988	8.59	7,436	0.0044
3	371,183	17.7	7,143	0.00229
4	192,916	38	6,728	0.00131
5	94,236	92.9	6,307	0.000776
6	40,046	176	5,911	0.000434
7	15,055	444	5,671	0.000246
8	5,186	851	5,787	0.000112
9	617	1100	6,344	0.000111

Table S2: Spatial variables used to predict methane concentrations.

Dataset	Variables	Spatial resolution	Temporal resolution	Type	Ref.
Global topography	Elevation, catchment slope	1 km	–	Physical	[2]
Worldclim 2.0 (version 1)	Temperature, precipitation	1 km	month	Climate	[3]
Global Reach scale A-priori Discharge Estimates	Discharge, river slope, gas transfer velocity	GRADES reach	month	Physical	[1]
MODIS gross primary production and net photosynthesis	Gross Primary production, Net primary production	1 km / 5 km	Year / month	Biological	[4]
Global gridded soil respiration	Heterotrophic and autotrophic respiration	55 km (0.5 degrees)	month	Biological	[5]
Harmonized world soil database	Soil properties	1 km	–	Soil	[6]
Global Land Cover	Fraction of multiple types of land cover	1 km	–	Land cover	[7]
Global Patterns of Groundwater Table	Depth to groundwater table	1 km	month	Soil	[8]
Global nutrient inputs into freshwater inputs	Nutrient inputs from multiple sources into rivers	55 km (0.5 degrees)	–	Soil	[9]
Fertilizer inputs	Fertilizer application in soils	10 km	–	Soil	[10]
Gridded population of the world	Population density	1 km	–	Human	[11]
Global Human Footprint maps	Global Human Footprint	1 km	–	Human	[12]
Peatland cover	Peatland cover	1 km	–	Land cover	[13]
Global aridity index database	Aridity and Runoff	1 km	month	Climate	[14]
Net Ecosystem Exchange from the ACOS GOSAT	Terrestrial Net ecosystem Exchange	110 km (1 degree)	month	Biological	[15]

Log-transformed variables included: CH_4 concentration, river discharge, catchment area, population density, river slope, soil organic carbon, soil $CaCO_3$ and $CaSO_4$ concentration, gas transfer velocity, groundwater table depth, fertilizer inputs, wetland land cover.

Table S3: Partitioning of uncertainty in the different parameters used to generated estimate of diffusive emissions, as well as assessment of other relevant processes and pathways that may create variability in these estimates.

Process	Internal variables	% internal uncertainty	Contribution to total uncertainty	Potential future improvements
Diffusive emissions	CH_4 concentration	49.9[a]	*medium*	More reach-scale measurements. Better world coverage across seasons (See figure S1)
	Gas transfer velocity	2.9[a]	*low*	Higher spatial resolution hydrological datasets and slope
	River area	47.2[a]	*medium*	Better upscaling of river area using remote sensing products
Ebullitive emissions			*high*	More reach-scale measurements Better understanding of broad-scale drivers.
Methane oxidation			*medium*	More reach-scale measurements

[a] Variance quantified within the Monte Carlo simulation. For each river reach, the results of the Monte Carlo simulation (n = 1,000) were analyzed using ANOVA, see methods for details. The values shown are global averages.

Table S4. Model performance statistics (R^2 on top and [RMSE] in brackets) of the three machine-learning algorithms tested, for each of the monthly datasets.

	**Jan**	**Feb**	**Mar**	**Apr**	**May**	**Jun**	**Jul**	**Aug**	**Sep**	**Oct**	**Nov**	**Dec**
Neural network	0.26 [2.5]	0.26 [2.54]	0.27 [2.3]	0.11 [2.72]	0.25 [2.43]	0.25 [2.58]	0.23 [2.28]	0.24 [2.48]	0.16 [2.25]	0.21 [2.32]	0.1 [2.68]	0.19 [2.71]
Random Forest	0.7 [1.73]	0.48 [1.81]	0.59 [1.93]	0.53 [2.04]	0.44 [2.24]	0.52 [2.07]	0.55 [1.95]	0.54 [1.94]	0.57 [1.79]	0.57 [1.88]	0.54 [1.85]	0.54 [1.96]
Gradient boosting	0.72 [1.65]	0.46 [1.80]	0.58 [1.82]	0.49 [2.15]	0.42 [2.1]	0.46 [2.28]	0.52 [1.87]	0.51 [2.03]	0.60 [1.88]	0.51 [1.88]	0.43 [2.09]	0.55 [2]

Any use of trade, firm, or product names is for descriptive purposes only and does not imply endorsement by the U.S. Government.

References:

1. Lin, P. *et al.* Global Reconstruction of Naturalized River Flows at 2.94 Million Reaches. *Water Resources Research* **55**, 6499–6516 (2019).
2. Amatulli, G. *et al.* A suite of global, cross-scale topographic variables for environmental and biodiversity modeling. *Sci Data* **5**, 180040 (2018).

3. Fick, S. E. & Hijmans, R. J. WorldClim 2: new 1-km spatial resolution climate surfaces for global land areas. *International Journal of Climatology* **37**, 4302–4315 (2017).
4. Zhao, M. & Running, S. W. Drought-Induced Reduction in Global Terrestrial Net Primary Production from 2000 Through 2009. *Science* **329**, 940–943 (2010).
5. Hashimoto, S. *et al.* Global spatiotemporal distribution of soil respiration modeled using a global database. *Biogeosciences* **12**, 4121–4132 (2015).
6. FAO, IIASA, ISRIC, ISSCAS & JRC. Harmonized World Soil Database - HWSD (version 1.2). (2012).
7. Tateishi, R. *et al.* Production of Global Land Cover Data – GLCNMO2008. *Journal of Geography and Geology* **6**, p99 (2014).
8. Fan, Y., Li, H. & Miguez-Macho, G. Global Patterns of Groundwater Table Depth. *Science* **339**, 940–943 (2013).
9. Beusen, A. H. W., Van Beek, L. P. H., Bouwman, A. F., Mogollón, J. M. & Middelburg, J. J. Coupling global models for hydrology and nutrient loading to simulate nitrogen and phosphorus retention in surface water – description of IMAGE–GNM and analysis of performance. *Geoscientific Model Development* **8**, 4045–4067 (2015).
10. Nishina, K., Ito, A., Hanasaki, N. & Hayashi, S. Reconstruction of spatially detailed global map of NH_4^+ and NO_3^- application in synthetic nitrogen fertilizer. *Earth System Science Data* **9**, 149–162 (2017).
11. Doxsey-Whitfield, E. *et al.* Taking Advantage of the Improved Availability of Census Data: A First Look at the Gridded Population of the World, Version 4. *Papers in Applied Geography* **1**, 226–234 (2015).
12. Venter, O. *et al.* Global terrestrial Human Footprint maps for 1993 and 2009. *Sci Data* **3**, 160067 (2016).

13. Melton, J. R. *et al.* A map of global peatland extent created using machine learning (Peat-ML). *Geoscientific Model Development Discussions* 1–44 (2022) doi:10.5194/gmd-2021-426.

14. Zomer, R. J., Xu, J. & Trabucco, A. Version 3 of the Global Aridity Index and Potential Evapotranspiration Database. *Sci Data* **9**, 409 (2022).

15. Jiang, F. *et al.* A 10-year global monthly averaged terrestrial net ecosystem exchange dataset inferred from the ACOS GOSAT v9 XCO_2 retrievals (GCAS2021). *Earth System Science Data* **14**, 3013–3037 (2022).
